# The nitrogen nutrition potential of arable soils

**DOI:** 10.1038/s41598-019-42274-y

**Published:** 2019-04-10

**Authors:** Claas Nendel, Dennis Melzer, Peter J. Thorburn

**Affiliations:** 1 Leibniz Centre for Agricultural Landscape Research, Eberswalder Straße 84, 15374 Müncheberg, Germany; 2 CSIRO Agriculture and Food, 306 Carmody Road, St Lucia, Qld 4067 Australia

## Abstract

Soils are an important source of nitrogen in many of the world’s cropping systems. Especially in low-input production systems, nitrogen release from soil organic matter turn-over is the major part of the crop’s nitrogen supply and research suggests that this process is significantly affected by changes in climate. The knowledge of the amount of nitrogen being accountable for crop nutrition is purely empirical in many production areas in the world and data as a foundation of global-scale climate change and food security assessments is scarce. Here we demonstrate that nitrogen mineralisation in general follows similar rules as for carbon, but with different implications for agricultural systems. We analysed 340 data sets from previously published incubation experiments for potential nitrogen mineralisation which covered a large range of soils and climate conditions. We find that under warm and all-year humid conditions the share of potentially mineralisable nitrogen in the soil’s total nitrogen is significantly smaller than in dry or temperate environments. We conclude that – despite relatively high soil nitrogen stocks – soil-borne nitrogen supply for crop production is very low in tropical and humid subtropical environments, which is a critical piece of information for global assessments of agricultural production and food security.

## Introduction

Low-input agriculture represents a considerable share of global food production, especially where global market produce is not accessible or affordable^1^. The production of such systems is low in absolute terms, however, the number of people relying directly on its output is still high; in many regions of the Global South it remains the predominant food supply. Global assessments of food security under future conditions face not only the challenge of data scarcity, but also limited process knowledge with respect to low-input agricultural systems and their management. One of the reasons for this is the vast variety of cropping systems, technical equipment and management strategies across the regions, in contrast to the very similar approaches that have emerged for mechanised, high-input agriculture.

Among all farmer-controlled input factors, nitrogen (N) has the second-largest impact on plant growth after water, and in many of the world’s cropping environments soil organic matter (SOM) mineralisation is the predominant source of N for the crop^2^. However, N release from SOM mineralisation is difficult to measure *in-situ* and only few attempts have been made to do so, using micro-lysimeters^3^ or field incubations of soil cores^4,5^. From incubation experiments scientists learned that fresh organic litter turn-over through micro-organisms is mainly governed by soil temperature and soil water content, representing the two most important environmental factors that soil-inhabiting micro-organisms respond to^6^. Temperature and water availability through precipitation and evapotranspiration are two major climate features that are expected to change significantly in many or the world’s agricultural areas until the end of the century, and direct effects on the SOM turn-over dynamics are likely to occur^7,8^. Assuming that SOM turn-over will follow the same rules as surface-deposited litter, these findings may lead to the assumption that SOM mineralisation is highest under warm and humid conditions. On the other hand, recent findings also confirm that SOM turn-over is strongly controlled by soil geochemistry and accessibility of SOM by micro-organisms; factors that have the potential to override temperature and moisture relations^9^. A systematic analysis of nitrogen release from SOM across different environments, however, does not exist. Such information is crucial for assessments of agricultural production and food security, especially when simulation models are employed to produce them. Currently, much focus is on using simulation models for crop physiology in such assessments to capture the impact of climate and atmospheric CO_2_ levels on crop growth and yield formation^10,11^, while soil processes and trade-offs between food security (yields) and climate change mitigation targets (e.g. soil C sequestration, greenhouse gas emissions) are rarely addressed until now.

In this paper we review existing literature on N mineralisation in agricultural soils in order to (i) find patterns across different environments and (ii) quantify the share of the SOM pool which actively contributes to mid-term N release to the benefit of crops. This information is a useful step towards understanding the nutrient-related yield gap across the globe^12^ and improve the representation in crop models of a background N supply from soils. The latter outcome will lead to improved model-based assessments of global crop yields^10,13,14^ and nutritive qualities^15^, where agro-ecosystem models are typically applied in a relatively high resolution across contrasting environments with only coarse soil information as input.

## Results

**Figure 1:**
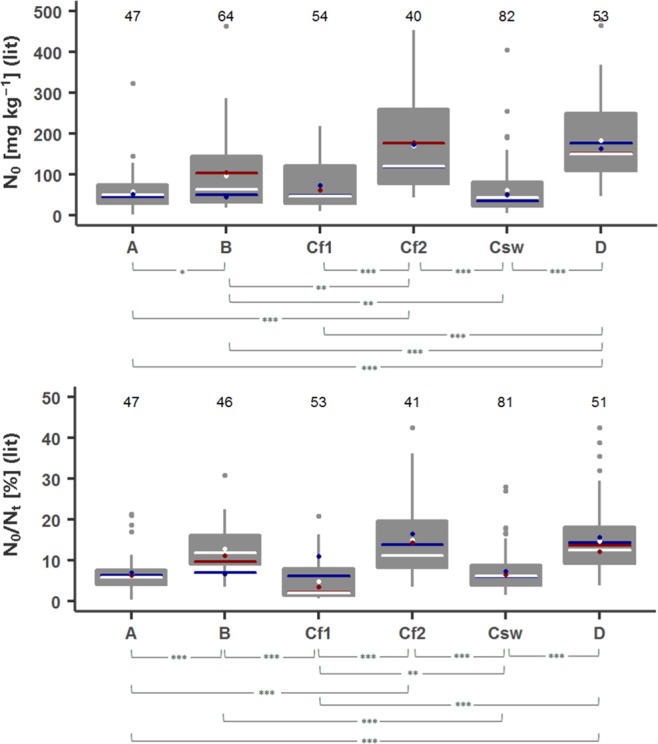
Boxplots for potential nitrogen mineralisation N_0_ and its share in total nitrogen (N_0_/N_t_) as observed and reported in literature, including the median (crossbars) and mean (dots) of the observed values (white), of the predicted values on the basis of the directly observed (red) and of the gridded data (blue). The analysis distinguishes humid tropical (**A**) dry (**B**) humid subtropical (**Cf**), further separated into warm (**Cf1**) and cool (**Cf2**) sub-groups, subtropical with dry periods (**Csw**) and temperate (**D**) climates. Thereby adjusted p-values < 0.0005 were marked with “***”, <0.005 with “**” and <0.05 with “*”.

The largest stocks of potentially mineralisable N were found in temperate (D: 151.0 mg N kg^−1^) and cool humid subtropical climate (Cf2: 121.5 mg N kg^−1^), while in all other climate groups the stocks were, on average, rather small (Csw: 44.6 mg N kg^−1^; Cf1: 47.4 mg N kg^−1^; A: 50.0 mg N kg^−1^; B: 64.3 mg N kg^−1^) with the dry climate (B) and the warm humid subtropical (Cf1) including also higher values, thus spanning a larger range than the tropical (A) and semi-dry subtropical (Csw) groups (Fig. 1). On the basis of the directly observed values, the statistical model performed well and only B and Cf2 were slightly overpredicted. Using the model that was built from the gridded data, only D was slightly overpredicted while all other means were predicted very well (Fig. 1).

The greatest proportion of total N that was potentially mineralisable N (N_0_/N_t_) was found in cool humid subtropical (Cf2: 11.28), temperate (D: 12.62) and dry climates (B: 11.83), while smallest shares are found in the warm humid subtropical (Cf1: 2.07), tropical (A: 5.92) and semi-dry subtropical (Csw: 6.19) climates (Fig. 1). Similar C to N ratios given, reactive N pool sizes correspond well with total SOM stocks for the first group, while for the second group medium-size SOM stocks and high organic matter input and turn-over rates result in only little amounts of plant-available mineral N being released. The statistical model based on the directly observed values performed very well in predicting the ratios, while the gridded data model slightly underpredicted the mean of the B group and slightly overpredicted the mean of the two Cf groups.

## Discussion

**Figure 2:**
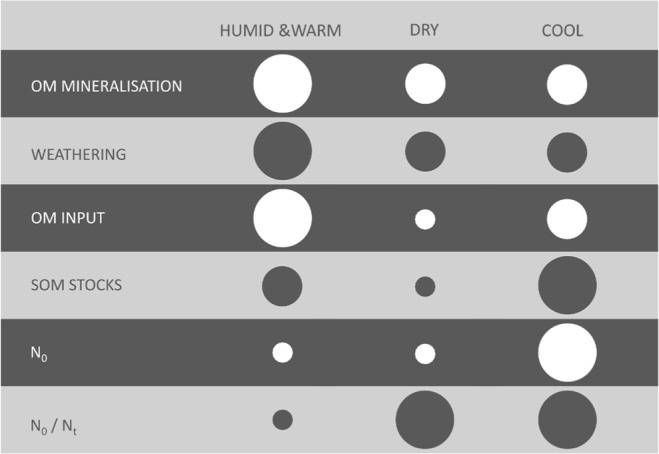
Schematic overview of dominating processes and system states across general climate characteristics, addressing fresh organic matter (OM) decomposition in terrestrial ecosystems^20,63^, bedrock weathering rates^64^, organic matter productivity and input to soils^16^, resulting soil organic matter (SOM) stocks, potentially mineralisable nitrogen (N_0_) from SOM as derived from incubation studies^37^ and the relation between N_0_ and the total organic nitrogen stock (N_t_).

Our results confirm earlier findings that TOC and N_t_ contents are largest in those climates (A, Cf1) where rainfall and temperature regimes allow strong biomass growth and facilitate correspondingly high organic matter input into the soil via litter fall, root exudates and residues^16^ or where decomposition is slowed down due to cooler temperatures while biomass growth is still moderately high (Cf2, D; Fig. 1). However, our results also show that high TOC (and N_t_) contents do not always correspond to high N_0_ as a proportion of N_t_ (i.e. N_0_/N_t_) across climates (Fig. 2). The difference in N_0_/N_t_ is likely to be a result of (i) SOM stabilisation mechanisms in soils^17^ and (ii) conditions that influence the soil micro-organisms in their ability and motivation to consume SOM and release N^18^. Both factors are directly and indirectly influenced by climate, of which “indirectly” refers to long-term weathering and soil formation processes under influence of climate and the resulting soil properties that govern the above-mentioned factors^19^.

Beside geochemistry, soil moisture and temperature are the most important variables influencing the decomposition of SOM^20^. In humid and warm conditions microbial activity is high, enabling the decomposer community to rapidly break-down and consume the large amounts of organic litter being produced^21^. However, biochemical weathering rates are also high, resulting in soils dominated by iron and aluminium hydroxides (some soil types like Arenosols or Vertisols being an exception), which have been observed as primary associates of SOC^22^. Forming stable metal-humus complexes and micro-aggregates, they protect organic matter against microbial decomposition^23–25^. With a high proportion of physically and biochemically stabilised SOC, also N is poorly available for microbial consumption, expressed as a small share of N_0_ in an else medium-sized pool of N_t_.

**Figure 3:**
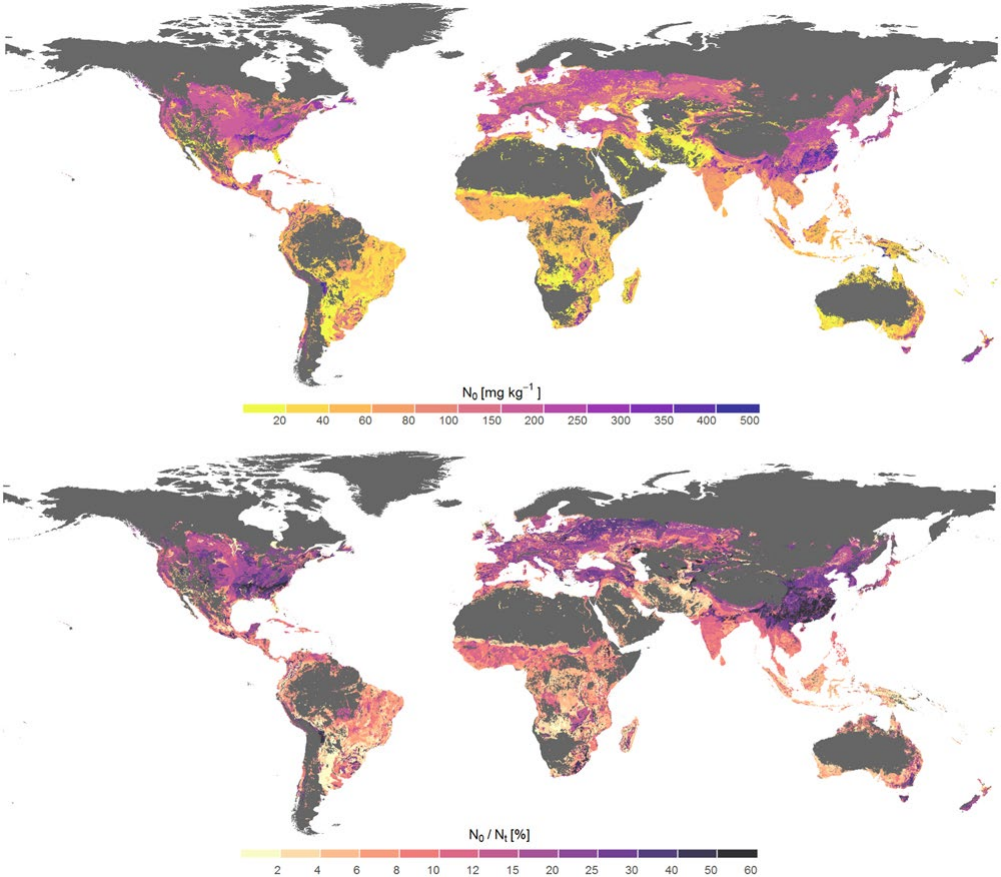
Global distribution of the nitrogen mineralisation potential of agricultural soils N_0_ (top) and its relation to soil organic nitrogen stocks, indicated by N_0_/N_t_ (bottom).

In subtropical dry and semi-dry regions soils are exposed to extremely variable climate. Soils in these areas host microbial communities adapted to frequent desiccation^26^, an attribute that seems to decline towards decreasing temperature environments^27^ and which explains why in dry and semi-dry regions fast decomposition rates prevail^28^. Together with reduced plant growth due to limited water supply and consequently low OM input into soil, this leads to generally small SOM stocks in these regions. However, since physical weathering dominates and soils developed under these climates exhibit lower potential to stabilise SOM, the mineralisable fraction remains high. Further, it is proposed that periodic drying and rewetting processes can enlarge the mobilisation of mineral associated organic matter^29,30^. Large mineralisable fractions of N_t_ also apply to soils of temperate climate zones^31^, but with a larger pool of SOM as a result of considerable biomass growth rates and decelerated SOM decomposition at lower temperature levels. Consequently, the total N mineralisation potential is highest in temperate climates (Figs 1 and 3).

**Figure 4:**
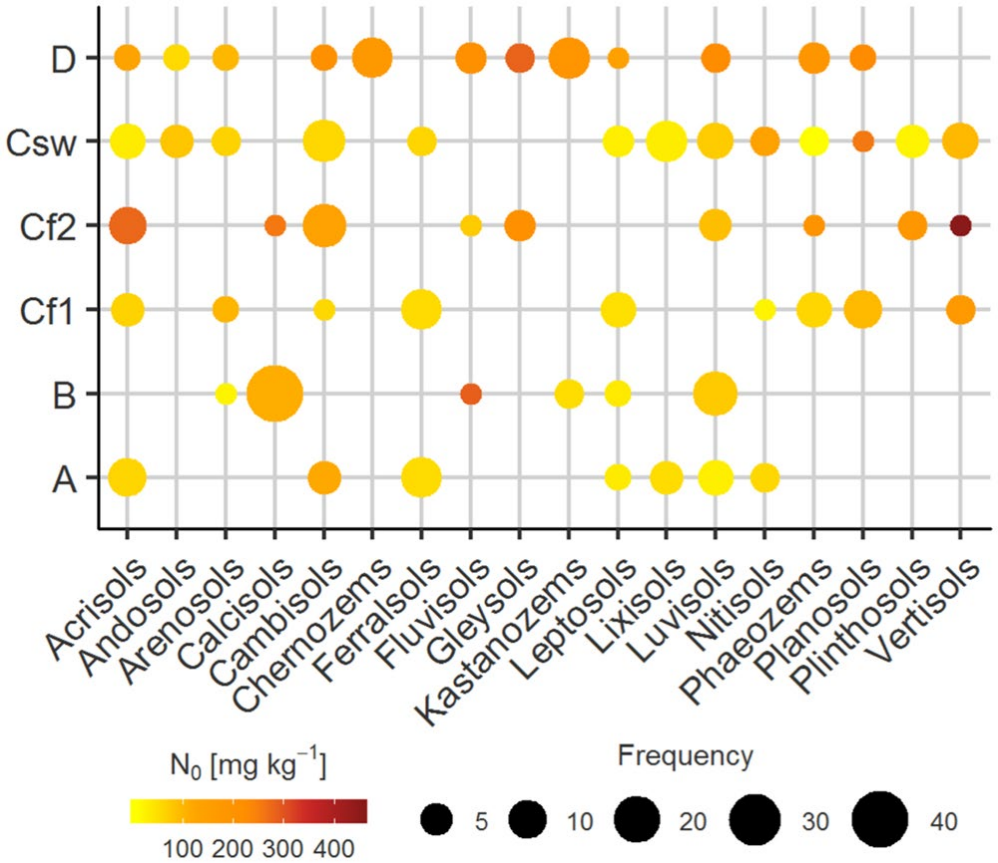
Representations of soil types in the nitrogen mineralisation data set. Size of the points represents the frequency of the soils in the literature data within each climate group. The colour scheme indicates the mean of N_0_ of the respective soil-climate group.

The models presented above were calculated using only a limited number of observations. It is for this reason that the range of each input parameter determines the lower and upper boundaries for the model equations (Supplementary Table S1). Using the equations for soils outside of these boundaries will deliver N_0_ estimates with higher uncertainties. Further, as shown in Fig. 4, only a limited number of soil types were found in the HWSD for the respective coordinates and the distribution of soils in the climate groups does only vaguely represent the natural occurrence of soil types within these climates. As a consequence, for some soils, the true value range is not well represented and a set of soil properties being atypical for the respective climate zone, e.g. for base-rich and fertile Vertisols from volcanic origin in the climate group A, will surely result in a strongly deviating N_0_ estimate. But also for other climate groups, distinct soil types show a unique nitrogen mineralisation potential, giving way to the assumption that other soil properties than those being available for the current analysis may further improve the N_0_ estimation (e.g. pH). Moreover, as only N_0_ data of arable soils was collected, the application of the models for soils under different land use will not be valid, as the land use has a strong influence upon the nitrogen dynamics of a soil^32^.

## Conclusion

In conclusion we confirm that climate fundamentally influences the soils mineralisation potential, by determining the organic matter input (assuming comparable management systems), controlling the temperature- and moisture- dependent microbial activity and directing soil development processes, thereby regulating the potential of soils to stabilise SOM and prevent its decomposition.

We provide a globally valid estimation of the N mineralisation potential of agricultural soils to feed both knowledge building in local agricultural practice for optimising fertiliser use and global assessments of agricultural production and food security. The N mineralisation potential of soils provides a proxy to inform agro-ecosystem simulation models when representing situations in which N fertiliser input is likely to remain below the crop’s demand and the additional N supply from fresh crop residues and SOM turns into sensitive regulators for crop yields.

## Methods

### General approach

We analysed 340 data sets from previously published incubation experiments which investigated potential nitrogen mineralisation (N_0_, see Eq. 1 below) in soils from a large range of climates. N_0_ describes the amount of soil organic matter that turns over to release N under optimum soil temperature and moisture within a few years. It is used here as a benchmark to compare across the very different data sets, bearing in mind that in-field N supply rates are additionally affected by the availability and quality of fresh organic matter (crop residues, organic amendments)^33^, soil disturbance processes (e.g. tillage)^34^ and the short-term dynamics of the micro-climatic conditions, to which micro-organisms respond differently as to constant laboratory environments^28,35^, and thus differ substantially from the rates determined through the N_0_ method. Present-day agro-ecosystem models, however, consider the effect of soil temperature and moisture and the presence of additional organic matter to predict soil-borne N supply to crops and they do very well if parameterised to the background N mineralisation rate of the soil^36^.

The only soil variables that were consistently supplied with N_0_ data in the literature were total organic carbon (TOC), total nitrogen content (N_t_), and soil texture. Across these studies, TOC and N_t_ contents were largest in soil from sites located in temperate climates, followed by tropical and all-year humid subtropical climates (Fig. 4). C to N ratios did not vary significantly across climates in the data set (median range: 10–12). N_0_ was largest at sites in temperate climates (D climate zone median: 151.0 kg N ha^−1^) and cool humid subtropical regions (Cf2: 121.5), with values that double those in dry regions (B: 64.3) and even triple those in tropical (A: 50.0), semi-dry (Csw: 44.6) and warm humid subtropical (Cf1: 47.4) climates.

The experiments were clustered into different climatic zones based on the locations of soils investigated. We then developed empirical relationships between N_0_ and various soil properties reported in the experiments within each climatic zone and, additionally, between N_0_ and soil properties reported in global soil databases within each climatic zone. The output of the latter was then used to develop a global map of N_0_ and of its share in total soil nitrogen N_t_, calculated as N_0_/N_t_.

### The nitrogen mineralisation potential

Comparing N mineralisation rates for different soils from literature sources requires a common standard, to which all data can be translated from their original form. Such a standard has been proposed by Stanford and Smith^37^, the mineralisation potential N_0_, which is the maximum amount of N being released from soil under optimum conditions for mineralisation by the microbial biomass (optimum temperature, soil water content, nutrient and oxygen supply). Their approach is to incubate small amounts of soil mixed with washed coarse sand at 35 °C and near-field capacity soil water contents, then fitting the following equation to the cumulative N release data to estimate N_0_:

1 $$N={N}_{0}\cdot (1-{e}^{(-k\cdot t)})$$

where *N* is the cumulative amount of nitrogen being released from incubated soil at any time *t*, *N*_*0*_ is the nitrogen mineralisation potential and *k* is the decomposition rate coefficient.

### Nitrogen mineralisation data

A literature review was carried out to collect data on nitrogen mineralisation potential of agricultural soils in different environments. Data on nitrogen mineralisation potential of soils relevant to this study is rare, as most of the recent publications examine the nitrogen release of soils amended with various kinds of residues and organic fertilisers^38–40^, concentrating on short-term nitrogen release rates^41^ or on non-agricultural soils^42,43^. However, a total of 340 data sets were extracted from 41 publications. The majority of the data (75%) were calculated values of N_0_ based on the incubation method of Stanford and Smith^37^. The remaining indicated some variation in the incubation methods used in the studies, such as the amount of soil incubated^44–46^, temperature and moisture regime during the incubations^47–49^ and/or duration of incubations^45,46,48,50^. All studies included in the analysis were evaluated for a sound estimation N_0_, especially when the incubation time seemed too short for the fitting of Eq. (1) to the time series^51^. Further, there were also differences in experimental approaches used in the studies, such as performing the incubations under anaerobic conditions^52^ or incubating the soil in polyethylene containers^39,53^. Data from these studies were included in the analysis, as these deviations from the standard procedure are assumed to have no substantial impact on the values of N_0_. This also applies to studies in which the influence of different management and tillage treatments on the N mineralisation dynamics was investigated. In these cases, the mean value of N_0_ across the different treatments was included e.g.^54–56^.

Additional explanatory information was obtained from the studies. This information included latitude, longitude, duration experiment, clay content, total organic carbon content (TOC) and total nitrogen content (N_t_). In many studies there was no information provided about basic soil characteristics, such as texture, TOC and N_t_, or about methods of their determination. If the latitude and longitude of original soil sampling was not stated within the publication, coordinates for the location were obtained from Google Earth^®^. Soil type representation across the dataset is summarised in Fig. 4.

### Definition of climate zones

**Figure 5:**
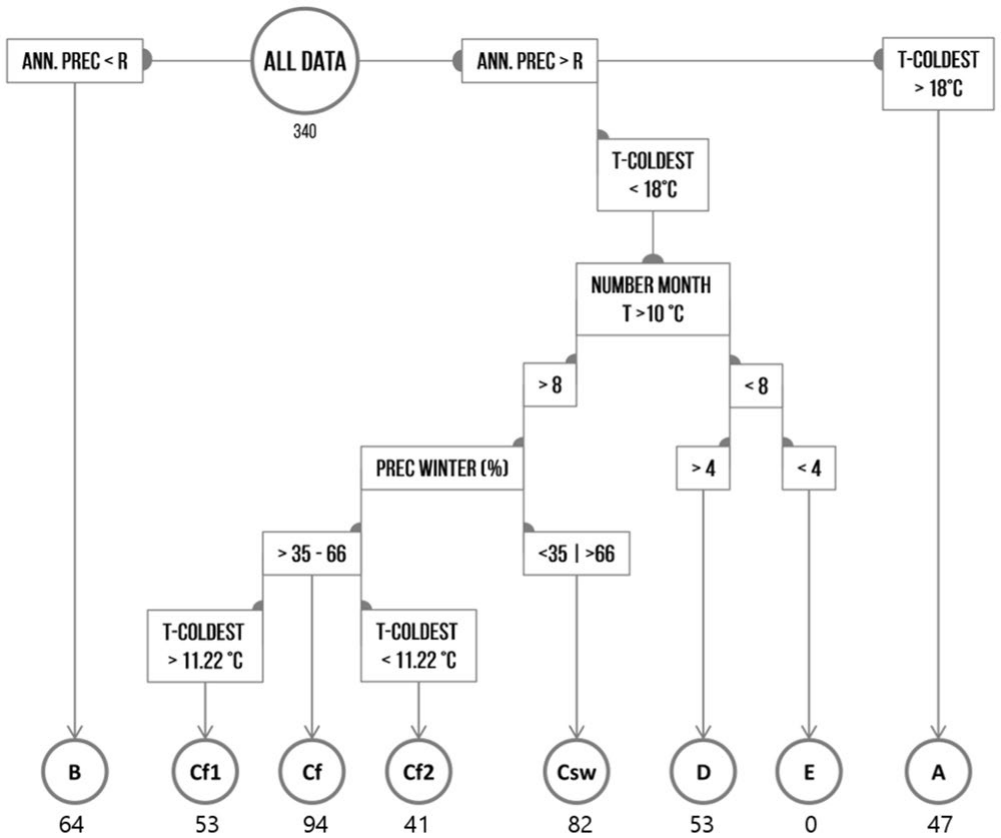
Climate grouping decision tree according to an adjusted Köppen-Trewartha scheme. Prec = precipitation, R = dryness threshold^58^, T = temperature. Numbers below the groups represent the sample size for each group respectively.

Climatic grouping was done using a cluster analysis on the Bioclim-CliMond data set in 30 arc seconds resolution^57^. The clusters corresponded well with the original Köppen-Trewartha scheme for A, B, Csw and D climates, which is why for the final grouping the Köppen-Trewartha scheme was applied (Supplementary Fig. S2). However, Cf climates (subtropical without dry season) formed two clusters with significantly different ratios of N_0_/N_t_ and annual coldest month. Accordingly, the decision tree was extended with a rule that identified subtropical climates without dry season as warm (Cf1: coldest month warmer than 11.22 °C) and cool (Cf2: coldest month cooler than 11.22 °C; Supplementary Fig. S2). For the calculation of the dryness threshold *R* the Köppen-Geiger equations were used (R = 2 · *T* + 14 for evenly distributed rainfall; =2 · *T* for regions with primarily winter rainfalls; =2 · *T* + 28 for regions with primarily summer rainfalls, where *R* denotes the mean annual precipitation threshold in centimetres and *T* the annual mean temperature in degrees Celsius). This approach was chosen since the differences between the Köppen-Geiger and the calculation preferred by Trewartha and Horn in 1980 was mainly based on imperial unit conversion^58^. Further the subdivision between humid subtropical (Cf) and semi-humid subtropical climates (Csw) refers to a differentiation of more than one third but less than two thirds of annual precipitation in the winter months (Cf) and vice versa (Csw). The number of data points in each climate group is well balanced, which ensured a non-biased analysis (Fig. 5).

### Determining nitrogen mineralisation potential from soil variables

Relationships between N_0_ and various soil properties (Table 1) were determined using the Eureqa software (Nutonian, version 0.98 beta^59^). Initially, a relationship was determined using data from all studies. However, the goodness-of-fit of the relationship to the data was poor (R^2^ < 0.1) so relationship were sought for data within each climate group. Models that included either TOC or N_t_ as input variable were selected from the Pareto front in the space spanned by complexity (number of coefficients) and accuracy (R^2^) as indicated by Eureqas’ internal Akaike Information criteria (AIC). However, to prevent overfitting only suggested equations with a complexity of <0.5·*n* were considered, where *n* denotes the size of the group. For the Csw group a higher complexity (0.6) was accepted as R^2^ was significantly smaller for less complex models.

**Table 1:** Gridded data extracted from the Regridded Harmonized World Soil Database (HWSD) and the Global Gridded Surfaces of Selected Soil Characteristics (IGBP-DIS) database (IGBP-DIS) as independent input variables for the derivation of statistical models.

HWSD		IGBP-DIS	
Variable	Unit	Variable	Unit*
Bulk density (BD)	*kg dm* ^− *3*^	Field capacity (FC)	*cm*
Topsoil organic carbon (TOC)	% weight	Plant-available soil water (PASW)	*cm*
Clay in topsoil (Clay)	% weight	Wilting point (WP)	*cm*
Silt in topsoil (Silt)	% weight	Total N (Nt)	*g m* ^− *2*^
Sand in topsoil (Sand)	% weight		
Gravel in topsoil (Gravel)	*% volume*		
CEC of the clay fraction (CEC)	*cmolc kg* ^− *1*^		

Abbreviations used in the following model descriptions (Supplementary Tables S2 and S3) are given in brackets. Note that not all variables were used in developing the empirical model.

*IGBP data refer to 100 cm soil depth.

Differences among climate groups and soil types were tested for significance using the non-parametric Kruskall-Wallis H-test, followed by a pairwise Wilcoxon Rank Sum Test (R - “pairwise.wilcox.test”), as the data was not normally distributed. Level of significance was set to 5%.

### Mapping the nitrogen mineralisation potential from agricultural soils at global scale

The global coverage of the literature data is too limited to extrapolate to the global scale at a useful resolution for further application of the N mineralisation potential in global assessments. For this reason we made an attempt to (i) include more soil properties and (ii) expand the applicability of N_0_ to points of interest for which no incubation experimental data is available in order. For this we used soil data from the Regridded Harmonized World Soil Database v1.2 (HWSD)^60^ and the Global Gridded Surfaces of Selected Soil characteristics database (IGBP-DIS)^61^ at a resolution of 5 × 5 arc-minutes (further referred to as “gridded soil data”; Supplementary Fig. S1) instead of the soil data given with the respective incubation experimental studies for creating a second set of Eureqa statistical models, assuming that at the given location both soils were the same (an assumption that has not been verified in the context of this analysis and will surely not hold for all data pairs).

The land use and land cover data of the HWSD for total cultivated land^62^ was used in 5 × 5 arc minutes resolution as the base raster for the generation of a global N_0_ map. Only grid cells with a cultivation area greater than zero were taken into account, as the models are only valid for arable soils. Subsequently, these cells were climatically grouped as described in the previous section. The gridded soil data was extracted for the respective cells and assigned to each literature N_0_ based on the location of the literature site. Models were generated using Eureqa as described above and N_0*pred*_ was calculated for each grid cell using the respective model of each climatic group and the gridded soil data as input. If literature reported neighbouring data points, the allocation to a grid cell would lead to the same TOC value for different N_0_. In such case, the deviation from the mean of the original TOC at the same location was used to correct the predicted TOC values to remain with the same number of data pairs. N_0_/N_t_ was then calculated from N_0*pred*_ and from N_t_ of the IGBP-DIS data in [g m^−3^] and transformed into [g kg^−1^] by using BD (for TOC values extracted from the gridded soil data see Supplementary Fig. S2).

Predicting N_0_ using gridded soil data (“predicted”) revealed a similar pattern across the climate zones as compared to the original soil data (“observed”) reported with the literature. A direct comparison of N_0_ predictions using the models created by using the respective basic soil data (TOC, N_t_, clay content) as Eureqa input demonstrated a good representation of the observed values by the predicted (Supplementary Fig. S3). Only for the temperate climate zone (D) a coefficient of determination <0.3 was found, where most of the high values of N_0_ observed in experiments are represented by much lower predictions, while at the lower end only a few mismatches were found. The coefficients of determination of the models developed on the gridded soil data were smaller than those developed on the data from the experiments, although greater than 0.28 in six of the seven climate groups.

## Supplementary information

Supplementary Information

## Data Availability

All data is available in the supplementary material or in a data repository to which a link is provided in the supplement.
