# Supplementary material for: The nitrogen nutrition potential of arable soils
Source: Sci Rep. 2019 Apr 10;9:5851. doi: 10.1038/s41598-019-42274-y (PMC6458312; doi:10.1038/s41598-019-42274-y)
Supplement: Supplementary file 1 — Supplementary Information [file 41598_2019_42274_MOESM1_ESM.pdf]

Supplementary Information for

The nitrogen nutrition potential of arable soils

C. Nendel, D. Melzer, P.J. Thorburn

Claas Nendel
Email: nendel@zalf.de

This PDF file includes:

- Supplementary text
- Figs. S1 to S4
- Tables S1 to S3
- References for SI reference citations

Other supplementary materials for this manuscript include the following:

Dataset: https://zcloud.zalf.de/index.php/s/aBqSqYaUHsbKOcQ

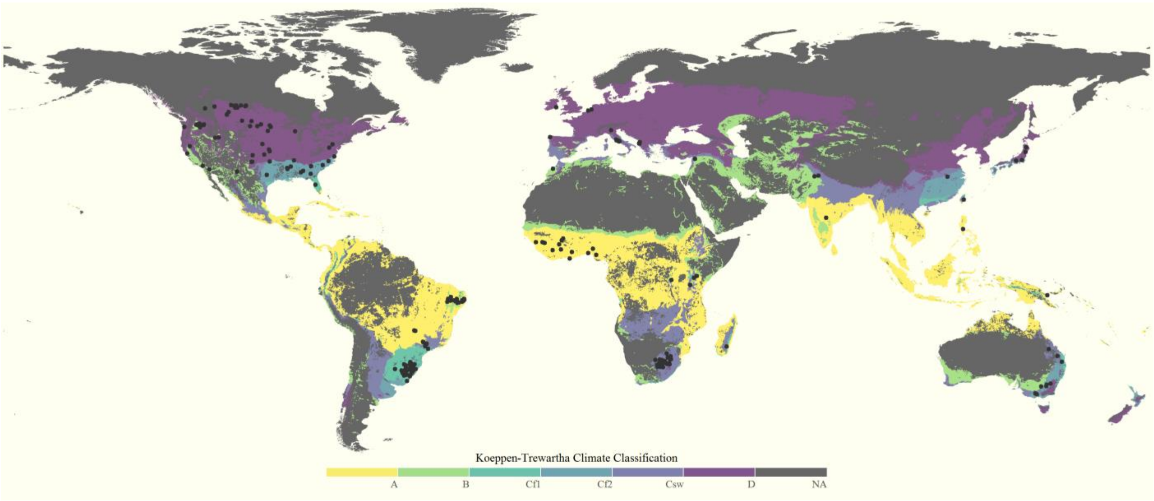

Fig. S1. Distribution of the soil data extracted from the Regridded Harmonized World Soil Database v1.2 1, for grid cells with more than 0% agricultural land. Cells were grouped according to the extended Köppen-Trewartha climate classification as described in the text. Sites from which soil was analyzed for potential nitrogen mineralization are plotted as black dots.

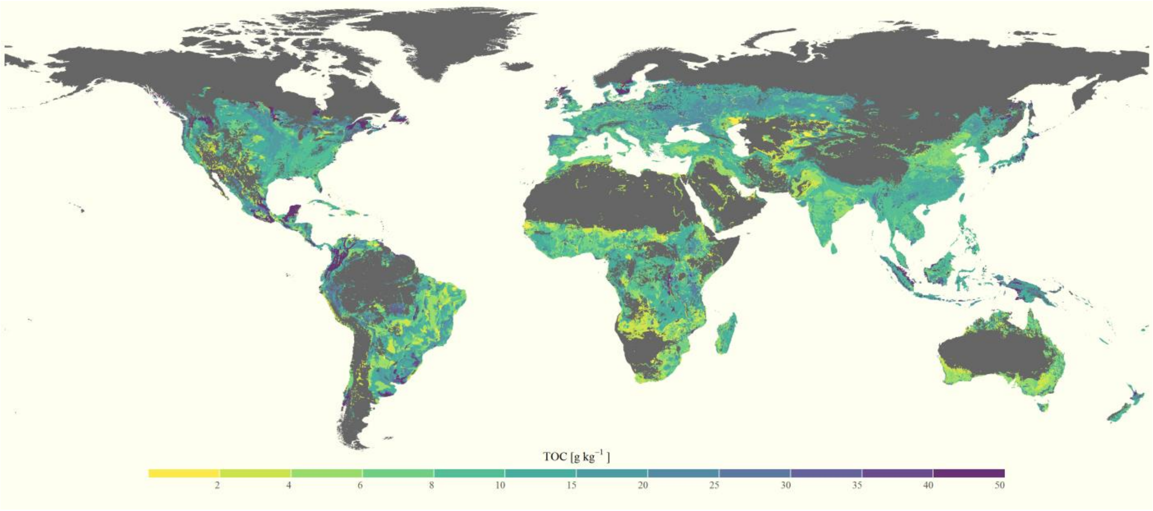

Fig. S2. Global distribution soil total organic carbon based on gridded data extracted from the Regridded Harmonized World Soil Database v.1.2 (1).

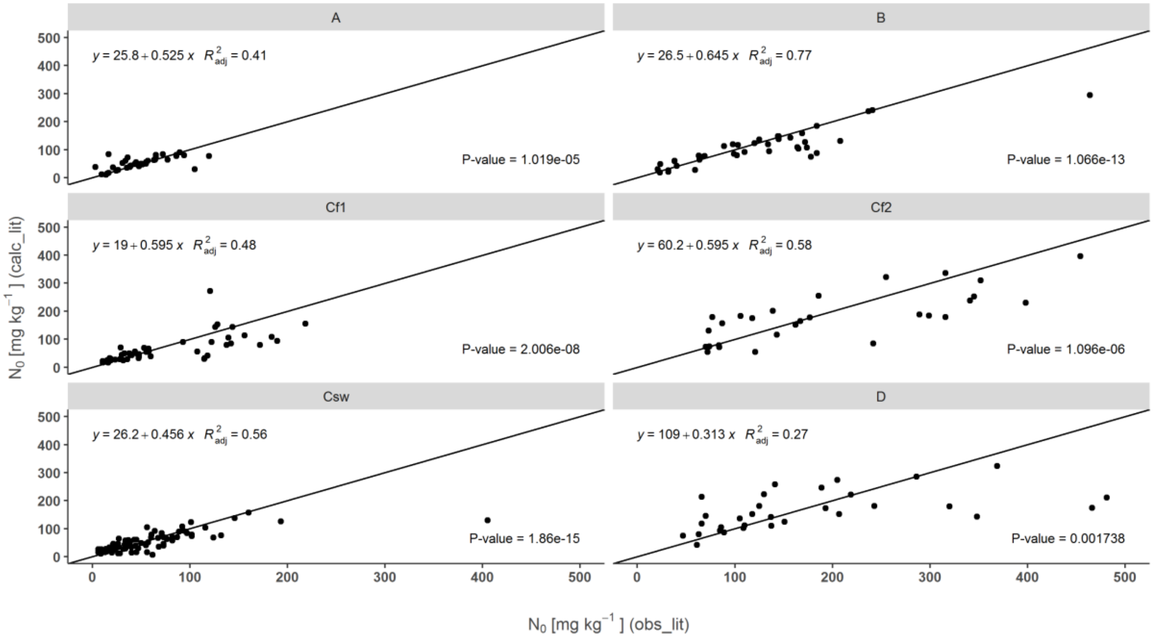

Fig. S3. Comparing literature data of N_0 with predicted values using the statistical models derived from the same data set for different Köppen-Trewartha Climate (KTC) zones.

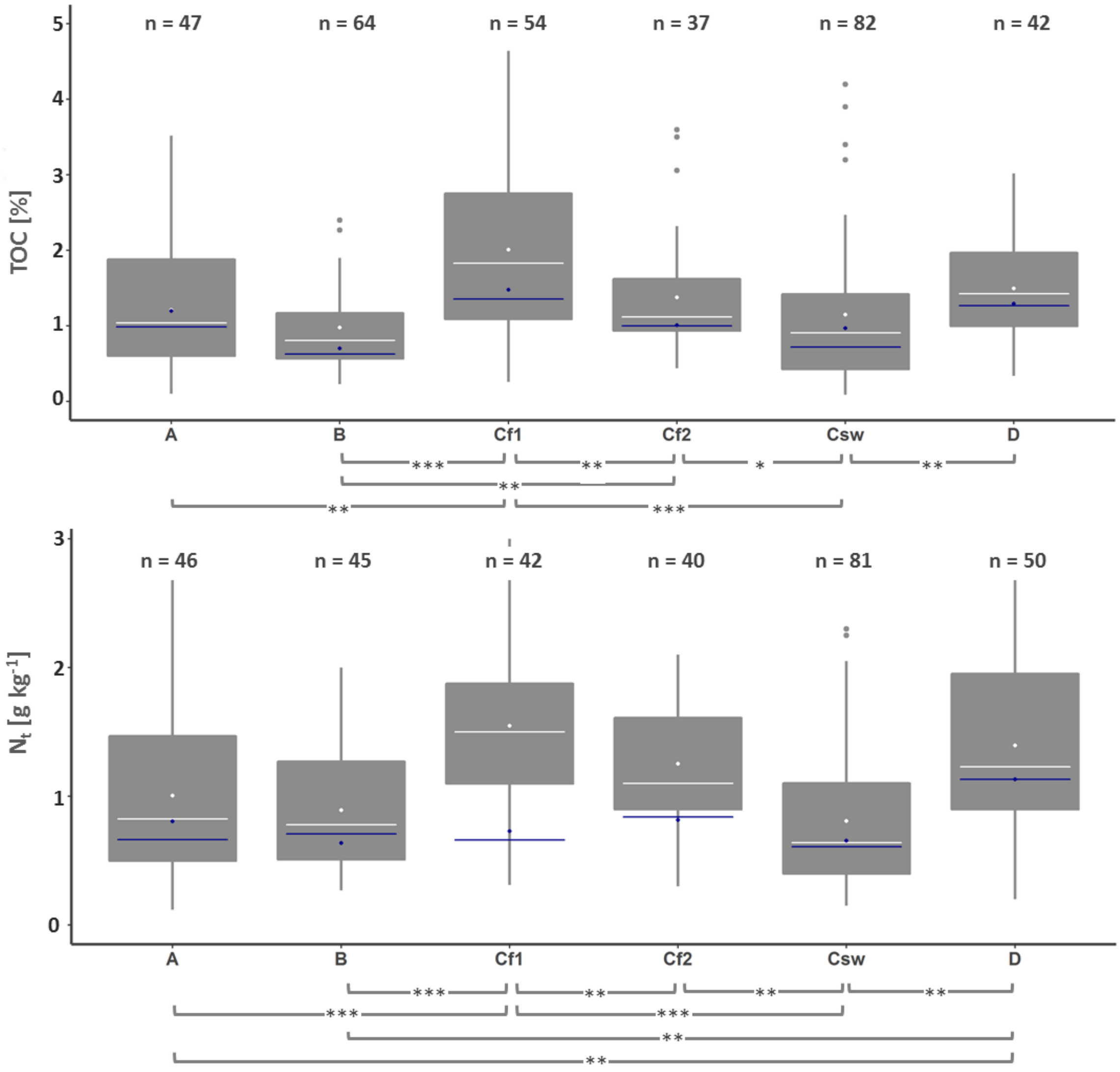

Fig. S4: Boxplots for total organic carbon (TOC) and total nitrogen (Nt) as observed and reported in literature, including the median (crossbars) and mean (dots) of the observed values (white) and of the predicted values on the basis of the gridded data (blue). The analysis distinguishes humid tropical (A), dry (B), humid subtropical (Cf), further separated into warm (Cf1) and cool (Cf2) sub-groups, subtropical with dry periods (Csw) and temperate (D) climates.

Table S1. Statistical models to explain the influence of total organic carbon (TOC), total nitrogen (N_t) sand (Sand) and clay content (Clay) on potentially mineralizable nitrogen (N_0). Models were derived from literature data using Eurequa.

Pred_obs	A	B	Cf	Cf1	Cf2	Csw	D
Model N_0=	$0.259 \cdot Clay + 1864.805 \cdot TOC / (37.556 + exp(1.322 \cdot TOC))$	$120.317 \cdot TOC + 78.806 \cdot TOC^2 + 0.098 \cdot Nt^2 \cdot Clay^2 – 24.312 – 6.233 \cdot TOC \cdot Nt \cdot Clay$	$111.315 + Nt \cdot Clay + 502.727 \cdot TOC / Lat- 1.615 \cdot Clay – 12.106 \cdot TOC$	$Clay \cdot log(TOC) + 123.738 \cdot Nt / TOC – 89.395$	$99.719 + 113.412 \cdot TOC + 0.045 \cdot TOC \cdot Clay^2 – 5.191 \cdot Clay –3.661 \cdot TOC^3$	$101.880 \cdot Nt+ 0.022 \cdot Clay^2 – Clay – Nt \cdot Clay$	$68.854 + 100.040 \cdot Nt – 0.033 \cdot Nt \cdot Clay^2$
R2	0.35	0.76	0.31	0.42	0.60	0.67	0.34
Correlation Coefficient	0.59	0.88	0.61	0.70	0.78	0.82	0.61
Mean absolute Error	16.54	20.72	36.94	26.13	52.90	17.02	53.33
Complexity	18	29	20	16	25	15	13
n	47	64	94	53	41	82	53

Table S2. Statistical models to explain the influence of total organic carbon (TOC), total nitrogen (N_t) sand (Sand) and clay content (Clay) on potentially mineralizable nitrogen (N_0). Models were derived from data extracted from the Regridded Harmonized World Soil Database v1.2 using Eurequa.

Pred_rast	A	B	Cf	Cf1	Cf2	Csw	D
Model N_0=	$29.058 + TOC \cdot WP + 0.070 \cdot Lat \cdot Silt \cdot Nt$	$3.824 + 1.014e^{-6} \cdot FC^5 \cdot Nt^2$	$0.785 + (17.210 \cdot silt + 0.001 \cdot Gravel \cdot Clay^3) / (Nt \cdot T\_coldest)$	$4.545 + exp (1.989 \cdot TOC) + exp (2.010 \cdot e^{-6} \cdot Sand \cdot FC \cdot Silt^2)$	$11.701 / TOC + 116672.037 \cdot TOC / (CEC \cdot PAWC)$	$TOC + Wp + 5.621 \cdot e^{-9} \cdot Clay \cdot Silt \cdot Sand \cdot Gravel \cdot Wp^4$	$0.155 \cdot Silt \cdot Sand + 2.900 / (2.195 – 2.461 \cdot TOC) – Lat$
R2	0.35	0.61	0.52	0.19	0.46	0.65	0.28
Correlation Coefficient	0.60	0.79	0.73	0.53	0.68	0.81	0.59
Mean absolute Error	19.17	23.64	40.88	39.50	51.13	17.34	54.28
Complexity	13	16	19	22	13	20	15
n	47	64	94	53	41	82	53

Table S3. Lower and upper boundary conditions of the data used for the generation of the models.

Variable	Unit	A		B		Cf1		Cf2		Csw		D	
Boundary		upper	lower	upper	lower	upper	lower	upper	lower	upper	lower	upper	lower
TOC [obs]	*% weight*	0.1	3.5	0.23	4.9	0.4	5.6	0.4	6.9	0.09	5	0.35	3.02
Nt [obs]	*$g\ kg^{-1}$*	0.1	3.8	0.27	3.3	0.5	4.2	0.3	4.7	0.15	2.3	0.39	3.1
Clay [obs]	*% weight*	5	68	7	76	0.8	80	1	77	3	78	7	60
BD [rast]	*$kg\ dm^{-3}$*	1.1	1.6	1.25	1.85	1.17	1.67	1.2	1.6	1.18	1.65	1.23	1.67
TOC [rast]	*% weight*	0.35	5.7	0.19	2.2	0.4	4	0.33	2.7	0.1	3.4	0.34	7
Clay [rast]	*$g\ kg^{-1}$*	8	75	2	47	6	62	10	56	7.8	70	6	49
Silt [rast]	*$g\ kg^{-1}$*	4	41	2	47	5	61	7	46	4	40	11	54
Sand [rast]	*$g\ kg^{-1}$*	13	85	2.6	96	6	89	17	83	18	85	18	83
Gravel [rast]	*% volume*	0	28	0	28	0	65	2	35	0	20	3	26
CEC [rast]	*$cmolc\ kg^{-1}$*	8	85	24	93	8	140	13	68	8.3	70	16	93
FC [rast]	*mm*	305	487	339	418	315	517	367	488	345	469	289	518
PASW [rast]	*mm*	165	266	200	270	177	270	183	295	180	289	174	303
WP [rast]	*mm*	138	250	114	187	138	255	118	253	71	251	83	222
Nt [rast]	*$g\ m^{-2}$*	0.37	1.9	0.26	1.04	0.36	1.43	0.33	2.11	0.23	1.78	0.46	1.94

References

1. Wieder WR, Boehnert J, Bonan GB, & Langseth M (2014) Regridded Harmonized World Soil Database v1.2. (Oak Ridge National Laboratory Distributed Active Archive Center, Oak Ridge, Tennessee, USA).
